# A Highly Potent and Broadly Neutralizing H1 Influenza-Specific Human Monoclonal Antibody

**DOI:** 10.1038/s41598-018-22307-8

**Published:** 2018-03-12

**Authors:** Aitor Nogales, Michael S. Piepenbrink, Jiong Wang, Sandra Ortega, Madhubanti Basu, Christopher F. Fucile, John J. Treanor, Alexander F. Rosenberg, Martin S. Zand, Michael C. Keefer, Luis Martinez-Sobrido, James J. Kobie

**Affiliations:** 1 Department of Microbiology & Immunology, University of Rochester, Rochester, NY USA; 2 Infectious Diseases Division, University of Rochester, Rochester, NY USA; 3 Division of Nephrology, University of Rochester, Rochester, NY USA; 4 Department of Microbiology, Informatics Institute, University of Alabama at Birmingham, Birmingham, AL USA

## Abstract

Influenza’s propensity for antigenic drift and shift, and to elicit predominantly strain specific antibodies (Abs) leaves humanity susceptible to waves of new strains with pandemic potential for which limited or no immunity may exist. Subsequently new clinical interventions are needed. To identify hemagglutinin (HA) epitopes that if targeted may confer universally protective humoral immunity, we examined plasmablasts from a subject that was immunized with the seasonal influenza inactivated vaccine, and isolated a human monoclonal Ab (mAb), KPF1. KPF1 has broad and potent neutralizing activity against H1 influenza viruses, and recognized 83% of all H1 isolates tested, including the pandemic 1918 H1. Prophylactically, KPF1 treatment resulted in 100% survival of mice from lethal challenge with multiple H1 influenza strains and when given as late as 72 h after challenge with A/California/04/2009 H1N1, resulted in 80% survival. KPF1 recognizes a novel epitope in the HA globular head, which includes a highly conserved amino acid, between the Ca and Cb antigenic sites. Although recent HA stalk-specific mAbs have broader reactivity, their potency is substantially limited, suggesting that cocktails of broadly reactive and highly potent HA globular head-specific mAbs, like KPF1, may have greater clinical feasibility for the treatment of influenza infections.

## Introduction

Although a licensed influenza vaccine has been available for over seventy years, influenza infections still remain a major public health concern. Annually, in the United States (US) influenza leads to ~15,000 deaths and ~300,000 hospitalizations, with ~3 to 5 million severe cases and 200,000 to 500,000 deaths per year globally^1–5^. In addition, the financial burden in the US averages more than 80 billion dollars annually, because of hospital costs or missed school or work days^6–8^. A key vulnerability is the need for annual selection of seasonal influenza vaccine composition to adequately match strains expected to be most prominent during the upcoming season. If the seasonal vaccine does not match the circulating strain the vaccine may be ineffective. Due to the propensity of influenza for antigenic drift and shift, and its tendency to elicit predominantly strain specific antibodies (Abs), humanity remains susceptible to waves of new strains with pandemic potential for which limited or no immunity may exist, as was the case in 1918 when the “Spanish Flu” killed ~30–50 million people^9^. Influenza A virus (IAV) contains 18 HA subtypes, which are further classified in two phylogenetic groups: group 1 (H1, H2, H5, H6, H8, H9, H11, H12, H13, H16, H17 and H18 subtypes) and group 2 (H3, H4, H7, H10, H14 and H15 subtypes). Currently, only influenza type A H1 and H3, and type B viruses are circulating in humans and are included in the seasonal vaccine. Recent pandemics, including the latest 2009 novel H1N1 pandemic^10,11^, which in less than 1 year infected more than 600,000 individuals worldwide causing nearly 16,000 deaths in over 200 countries^12^, demonstrate the need to develop new vaccine strategies and therapeutics that confer broad protection against diverse influenza strains.

The current anti-viral treatments (e.g. oseltamivir/Tamiflu, amantadine/rimantadine) for influenza are sub-optimal with increasing incidence of resistance and a limited therapeutic window (must start <48 h after symptom onset)^13–15^. Subsequently new preventive and therapeutic interventions for influenza are being sought. Monoclonal Abs (mAbs) continue to be a growing class of drugs in-part due to their high degree of specificity, limited off-target effects, and favorable safety profile^16–18^. In addition to their use in treatment of cancer and autoimmunity, several mAbs are already licensed or in clinical trials for the treatment and prevention of various infectious diseases, including the use of palivizumab for the prevention of Respiratory Syncytial Virus (RSV) infection^19,20^.

Human mAbs (hmAbs) have been isolated that have the ability to neutralize diverse influenza strains. These all target the hemagglutinin (HA) protein expressed on the surface of the virion and include, for instance hmAbs such as 5J8^21^, 1F1^22^ and CH65^23^, which bind multiple H1 isolates; hmAbs such as F10^24^ and CR6261^25^, which recognize all group 1 viruses; hmAbs such as CR8020^26^ and 2F04^27^ which recognize most group 2 viruses; hmAbs FI6/MEDI8852^28,29^, 2B06^27^, S6-B01^27^, 3I14^30^, and VS140^31^, which each recognize both group 1 (e.g. H1, H2, H5) and group 2 (e.g. H3, H7) viruses; or hmAb CR9114^32^, which recognizes both type A and type B viruses. Several of these hmAbs are currently in clinical trials and their characterization has led to the identification of conserved epitopes in influenza HA that might be valuable as targets for the development of universal influenza vaccines and/or therapeutics. mAbs with the greatest breadth consistently target the HA stem region, while those targeting the globular head of HA frequently are confined to only subtype-specific breadth^33–35^. The ability to isolate broadly reactive influenza hmAbs indicates that the human B cell response is capable of generating such a valuable response, the challenge now lies in inducing these responses in sufficient frequency, magnitude, and durability to confer broad protection.

Peripheral blood plasmablast B cells are an easily accessible population of cells that are highly enriched for specificity to insulting antigen or pathogen, which has made them highly useful for obtaining mAbs. In an effort to identify HA epitopes that if targeted may confer universally protective humoral immunity as well as generate hmAbs that may have broad spectrum activity, we examined plasmablasts from a subject that was immunized with the 2014–2015 seasonal inactivated influenza vaccine. Using deep immunoglobulin repertoire sequencing and single-cell immunoglobulin cloning we isolated and characterized a hmAb (KPF1) with broad and potent neutralizing activity *in vitro* against H1 influenza viruses, including 1918 influenza. Importantly, KPF1 was able to protect mice prophylactically against multiple H1 influenza strains and therapeutically against a lethal challenge with A/California/04/2009 H1N1 (pH1N1). KPF1 binds with high affinity to a novel conserved epitope region of the HA globular head, which is different than other previously described cross-reactive H1 mAbs, and represents an excellent option for the development of antiviral therapies based on Ab cocktails with broadly reactive and highly potent HA globular head-specific hmAbs for the treatment of H1 influenza infections in humans.

## Results

### Generation of KPF1 hmAb

**Figure 1:**
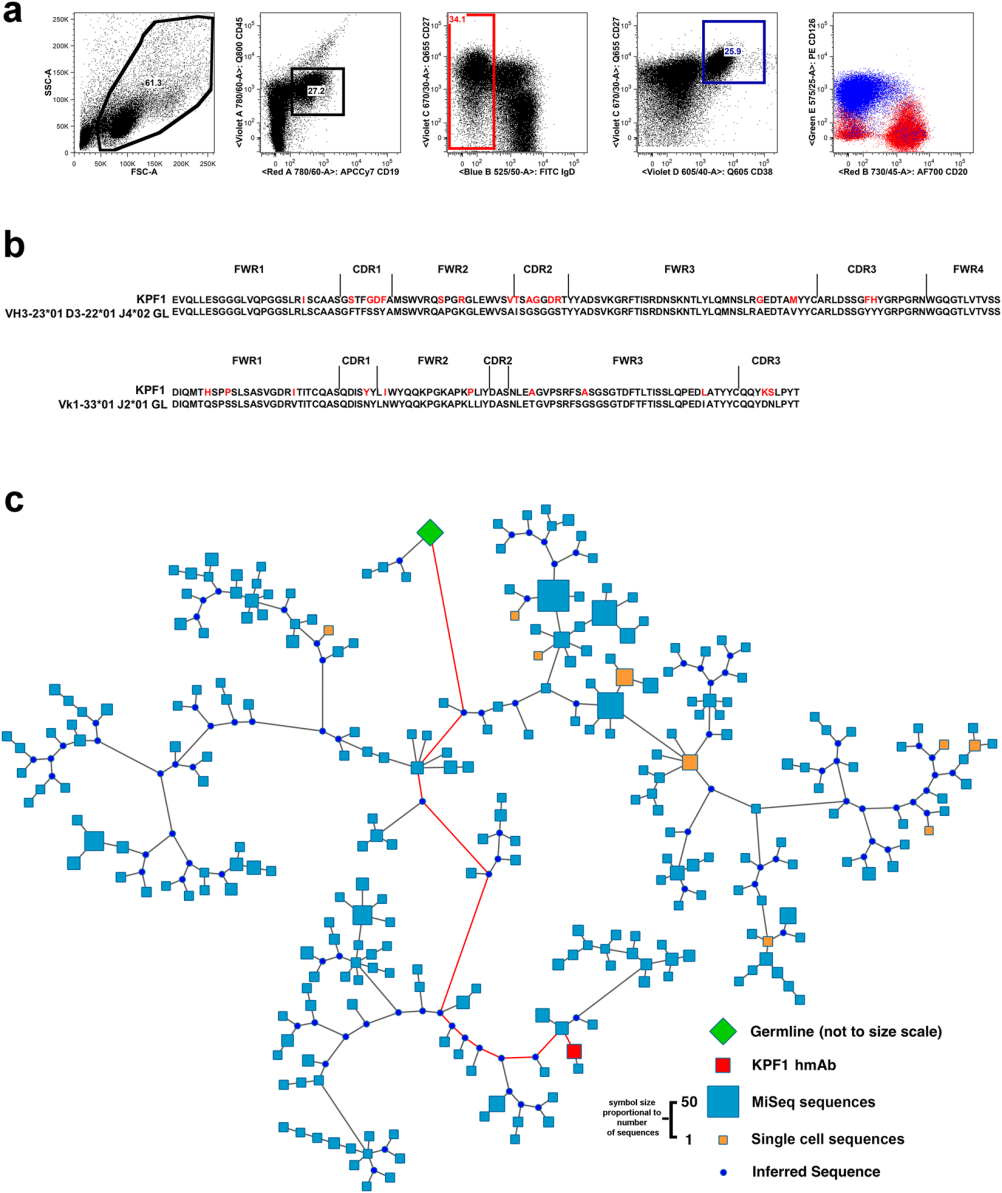
Isolation and molecular characterization of KPF1 hmAb. (**a**) Gating strategy to isolate peripheral blood plasmablasts (CD19 + IgD-CD38 + CD27++) 7 days after immunization. (**b**) Alignment of KPF1 VH and Vk with presumed germline amino acid sequences. (**c**) Phylogenic analysis of KPF1 lineage members based on amino acid sequence. Lineage members defined as same heavy chain V and J gene usage, HCDR3 length, and ≥85% HCDR3 similarity. Germline sequence is represented by green diamond, sequences obtained from single-cell plasmablast sequencing are represented by orange squares, the KPF1 mAb sequence is represented by the red square, sequences obtained by MiSeq-based deep sequencing of bulk total B cells are represented by blue squares, inferred intermediate sequences are represented by blue circles. Red line indicates the inferred pathway from germline to KPF1 hmAb. Size of symbols are proportional to the number of identical sequences obtained of an individual lineage member (N = 1–55), with the exception of the germline and KPF1 mAb sequences.

Peripheral blood plasmablasts (CD19 + IgD-CD38 + CD27++) were single cell sorted from a healthy subject seven days after immunization with the 2014–2015 seasonal inactivated quadrivalent influenza vaccine. This subject had a robust plasmablast response comprising ~25% of the IgD- compartment and that were predominantly CD20-CD126(IL-6Rα)+ (Fig. 1a), consistent with an antigen-specific response^36^. The plasmablasts were subjected to single-cell RT-PCR and sequencing of the immunoglobulin variable regions, and the dominant B cell lineage, comprising ~10% of the isolated plasmablasts was identified and is encoded by the immunoglobulin (Ig) heavy chain VH3–23 and kappa light chain Vk1–33 genes. Due to the dominance of this lineage among the plasmablasts, a representative member of the lineage was cloned as an IgG1 to generate the KPF1 fully hmAb. The heavy chain variable region (VH) contained 14% amino acid (8% NT) and kappa light chain variable region (V_k_) 10% amino acid (5% NT) mutations from the germline, consistent with affinity maturation (Fig. 1b). Subsequent deep sequencing of the B cell immunoglobulin repertoire at the same time point identified additional members of this lineage, including members with intermediate levels of somatic hypermutation, and those that had acquired additional mutations (Fig. 1c), suggesting the KPF1 hmAb developed after an extensive affinity maturation process.

### *In vitro* activity of KPF1

**Figure 2:**
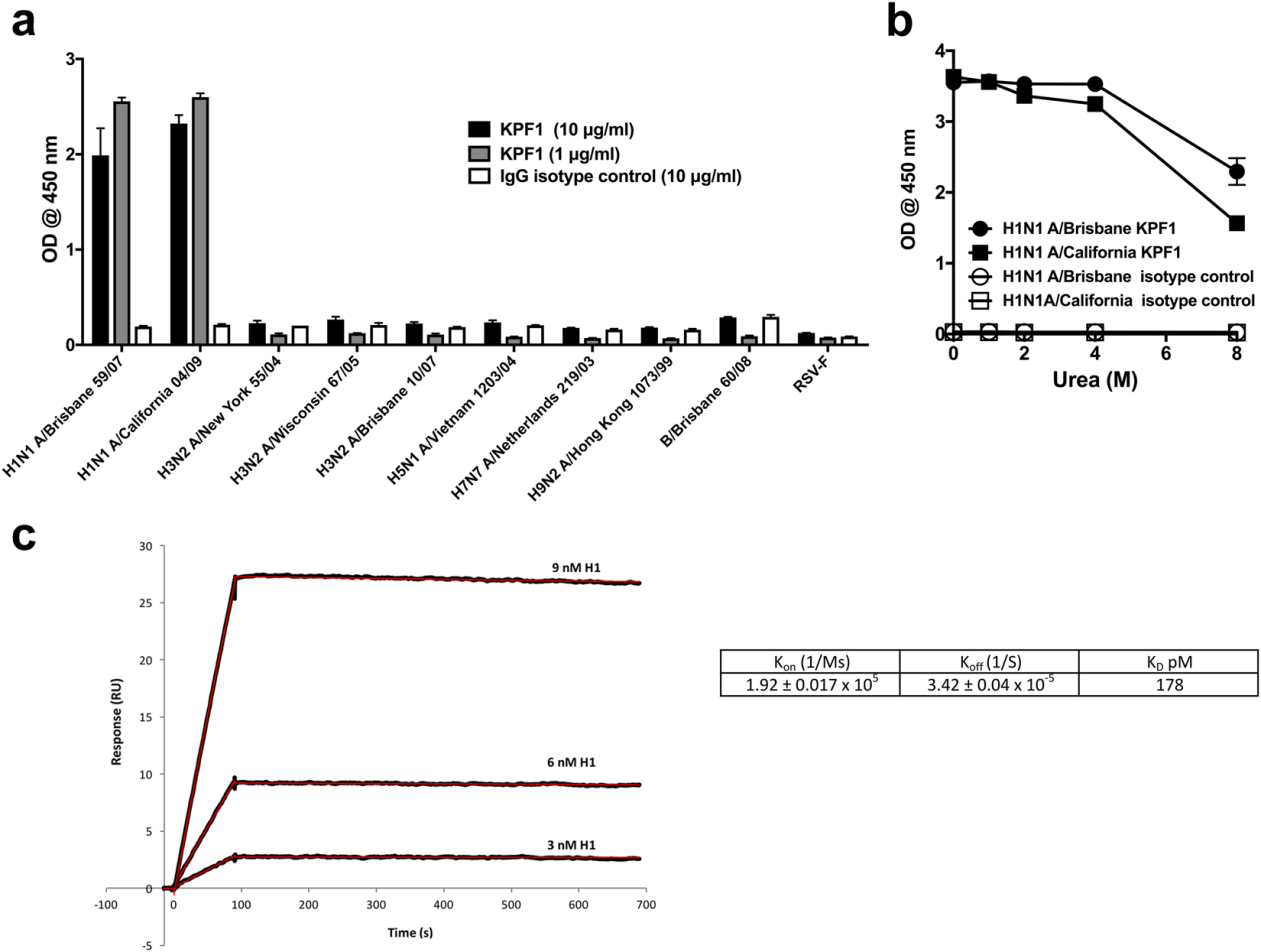
KPF1 hmAb is highly specific for H1 influenza. KPF1 hmAb and IgG isotype control hmAb were tested by ELISA for binding to (**a**) diverse recombinant influenza A H1 (A/Brisbane/59/07 and A/California/04/09), H3 (A/New York/55/04, A/Wisconsin/67/05 and A/Brisbane/10/07), H5 (A/Vietnam/1203/04), H7 (A/Netherlands/219/03), H9 (A/Hong Kong/1073/99) and influenza B (B/Brisbane/60/08) HAs and negative control protein (RSV-F) and (**b**) H1 (A/Brisbane/59/07 and A/California/04/09) HA proteins in increasing concentrations of urea. Symbols represent triplicate ± SEM. (**c**) Purified KPF1 was captured on a Protein G chip with the pH1N1 HA at decreasing concentrations passed over each channel. The data points are shown in black and the fit to a 1:1 binding model are shown in red. The results of one representative experiments of two are presented.

**Figure 3:**
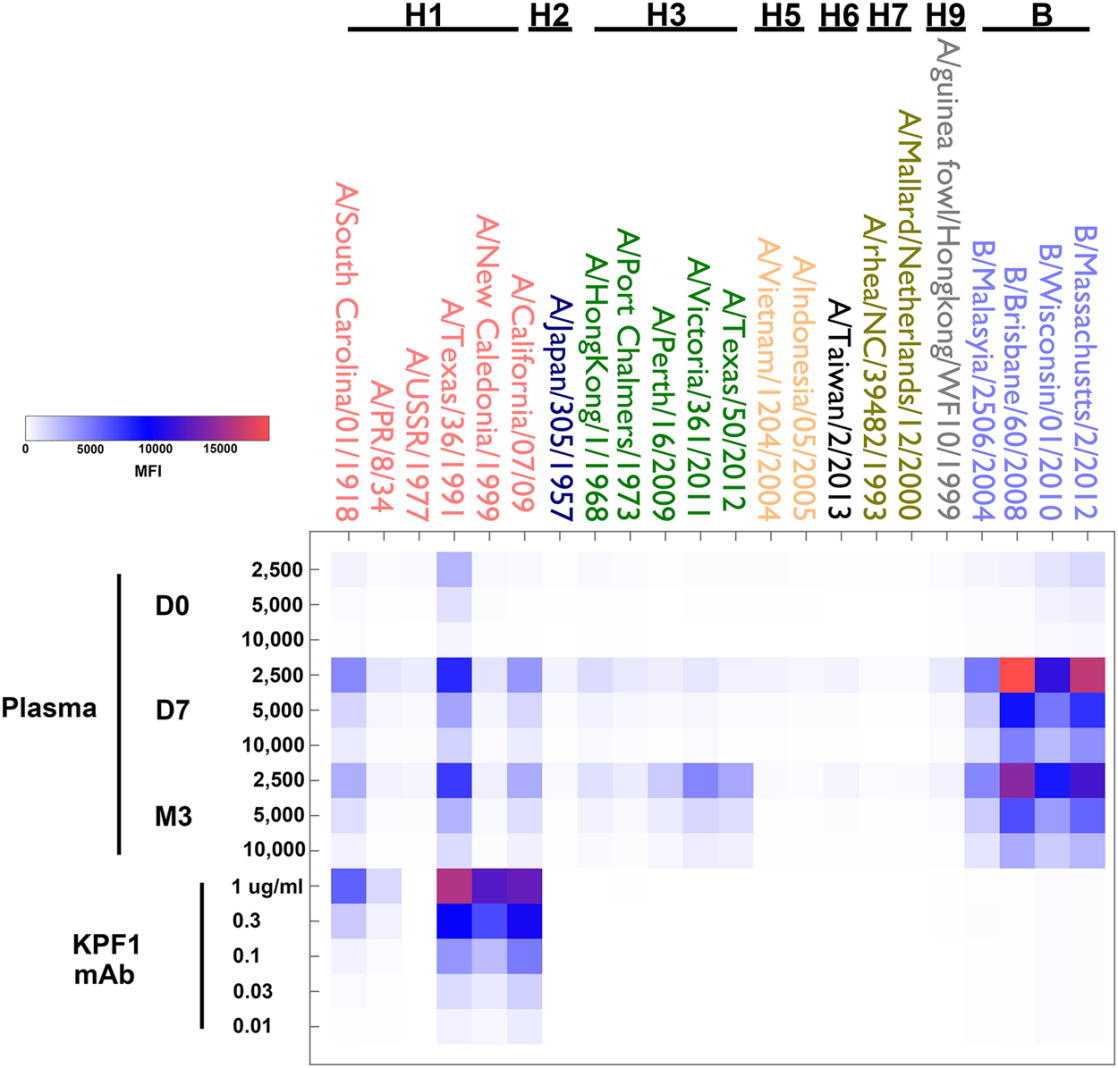
mPLEX-Flu binding profile. The patient’s plasma from before immunization (D0) and 7 days (D7) and 3 months (M3) post-immunization, and KPF1 hmAb were tested in decreasing concentration by multiplex assay for binding to the indicated recombinant influenza A H1, H2, H3, H5, H6, H7, H9 and influenza B HA proteins.

Initial characterization by ELISA revealed that KPF1 was highly specific for influenza H1 HAs, with no reactivity against influenza H3 or B HAs (Fig. 2a). Most of its binding (~50–70%) to HA was maintained even in 8 M urea, indicating high avidity (Fig. 2b). Surface plasmon resonance (SPR) determined KPF1 bound pH1N1 HA with very high affinity, the equilibrium dissociation constant (KD) of binding was 178 pM (Fig. 2c). The comprehensive binding profile of KPF1 against 23 HAs was determined by the fluorescent-bead based mPLEX-Flu assay that we have previously described^37^. KPF1 bound only to H1 HAs, including A/South Carolina/01/1918 H1N1 with a trend of greater reactivity to more recent H1N1 strains (Fig. 3). KPF1 bound 5/6 H1 HAs, failing to recognize A/USSR/1977 H1N1 at 1 μg/ml. The H1 reactivity profile for KPF1 mirrored the subject’s plasma Ab response, including its strong reactivity against TX H1N1, which dominated the subject’s pre-vaccination (D0) plasma response.

**Figure 4:**
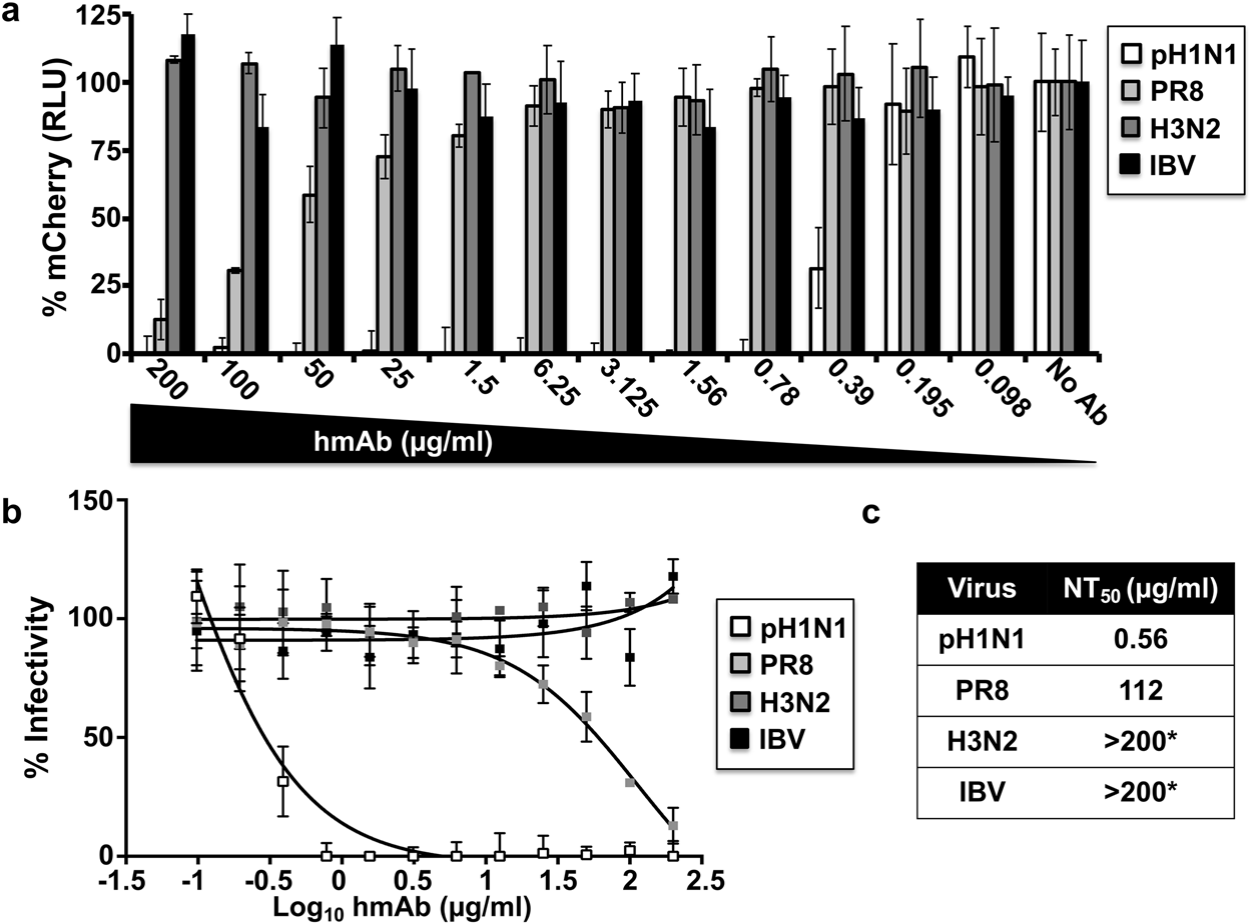
Potent *in vitro* neutralizing activity of KPF1 hmAb. Virus neutralization was determined using a fluorescent-based microneutralization assay^38,39^. MDCK cells were infected with mCherry-expressing pH1N1, PR8 H1N1, H3N2 and IBV, which were pre-incubated with two-fold serial dilutions of KPF1 hmAb. At 24 h p.i., virus neutralization was evaluated and quantified using a fluorescence microplate reader (**a**), and the percentage of infectivity calculated using sigmoidal dose response curves (**b**). Mock-infected cells and viruses in the absence of hmAb (No hmAb) were used as internal controls. Percent of neutralization was normalized to infection in the absence of hmAb. Data show means ± SD of the results determined for triplicates. (**c**) NT_50_ of KPF1 hmAb by fluorescent-based assay. *Highest amount of hmAb without detectable neutralizing effect.

In addition, using a fluorescent-based^38,39^ or a traditional microneutralization assay (Fig. 4 and Table 1, respectively), KPF1 was able to neutralize broad range of H1 viruses, including potent neutralization of pH1N1 (NT_50_ = 0.56 μg/ml Fig. 4; 1.46 μg/ml Table 1), NC H1N1 (NT_50_ = 4.56 μg/ml Table 1), and TX H1N1 (NT_50_ = 1.26 μg/ml Table 1), while neutralization of PR8 H1N1 was less efficient (NT_50_ = 112 μg/ml Fig. 4; 145.6 μg/ml Table 1). Neutralization of influenza A H3 or B strains was not detected even at the highest concentration of KPF1 tested (200 μg/ml) (Fig. 4a–c and Table 1). KPF1 also exhibited hemagglutination inhibition (HAI) activity against pH1N1 (Table 1). Overall KPF1 is specific to influenza A H1, and recognizes and functionally inhibits a wide range of H1 influenza virus isolates *in vitro*.

**Table 1:** Microneutralization and HAI assay.

Virus^(a)^	NT_50_ (µg/ml)^(b)^	HAI (µg/ml)^(c)^
pH1N1	1.46	0.78
TX H1N1	1.26	0.78
NC H1N1	4.56	3.12
PR8 H1N1	145.6	50
H3N2	>200*	ND
IBV	>200*	ND

^(a)^Viruses used in this assay: A/California/04/09 H1N1 (pH1N1), A/Texas/36/91 H1N1 (TX H1N1), A/New Caledonia/20/99 H1N1 (NC H1N1), A/Wyoming/3/03 H3N2 (H3N2), A/Puerto Rico/08/34 H1N1 (PR8 H1N1), or B/Brisbane/60/08 (IBV).

^(b)^MDCK cells were infected (100 PFU) with the indicated viruses, which were pre-incubated with 2-fold serial dilutions (starting concentration of 200 µg/ml) of the hmAb KPF1. At 48–72 h p.i., cells were stained with crystal violet and the NT_50_ was determined using sigmoidal dose response curves. Mock-infected cells and viruses in the absence of hmAbs were used as internal controls. *Highest amount of hmAb without detectable neutralizing effect.

^(c)^HAI assays were performed using 2-fold serial dilutions (starting concentration of 200 µg/ml) of KPF1 and 4 hemagglutinating units (HAU) of the indicated viruses. HAI titers were determined by adding 0.5% turkey RBCs to the virus-hmAb mixtures and defined as the minimum amount of hmAb that completely inhibited hemagglutination.

### *In vivo* activity of KPF1

**Figure 5:**
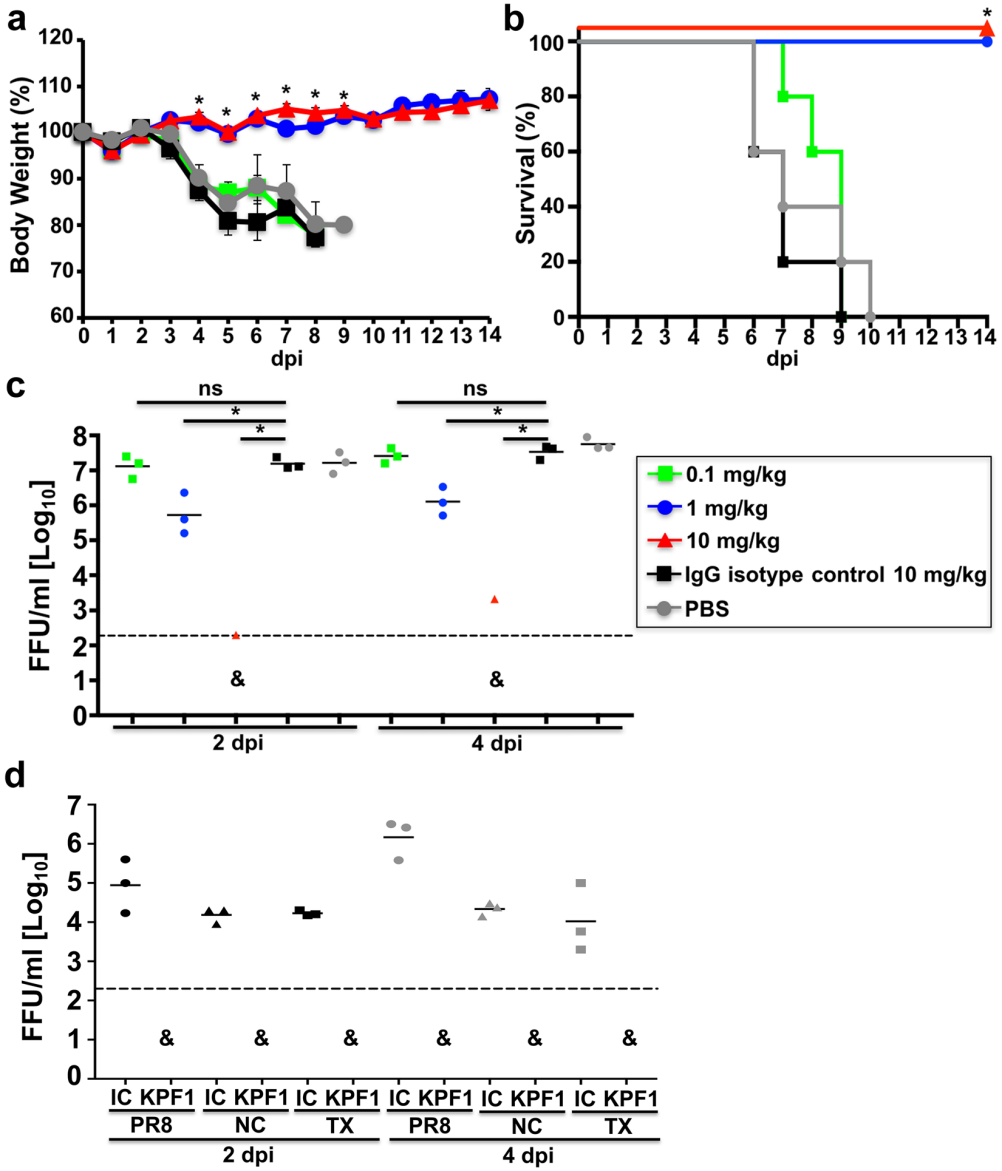
KPF1 hmAb restricts pH1N1 replication *in vivo*. Female C57BL/6 mice (N = 11) were treated i.p. with 0.1, 1 or 10 mg/kg of KPF1 hmAb, or with 10 mg/kg of an isotype control (IgG isotype control), or PBS 24 h before infection. Mice were then challenged with 10x MLD_50_ of pH1N1 and monitored daily for 2 weeks for body weight loss (**a**) and survival (**b**). Mice that lost 25% of their body weight were sacrificed. Data represent the means ± SD (N = 5). *Indicates p < 0.05 (when the differences between mice treated with 1 or 10 mg/kg of KPF1 hmAb and mice treated with PBS or IgG were significant using a one-tailed Student’s t test (body weight) or Mantel-Cox test (survival). To evaluate viral replication in the lungs (**c**), mice were sacrificed at 2 (N = 3) and 4 (N = 3) days p.i. and whole lungs were used to quantify viral titers by immunofocus assay (FFU/ml). *Indicates p < 0.05 using one-way ANOVA and Dunnett’s test for multiple comparison correction. Ns, no statistically significant differences. **(d)** Evaluation of KPF1 for its prophylactic activity against multiple H1 influenza strains. Female C57BL/6 mice (N = 6) received 10 mg/kg of KPF1 or IgG isotype control (IC) 24 h before viral infection. Mice were then challenged with 10x MLD_50_ of PR8 H1N1 (circles), TX H1N1 (squares), or NC H1N1 (triangles) and viral replication in lungs at 2 (N = 3) and 4 (N = 3) days p.i. (black and grey symbols, respectively) was evaluated as indicated above. For (**C)** and (**D)**, symbols represent data from individual mice. Bars, geometric mean lung virus titers; dotted line, limit of detection (200 FFU/ml). & indicates virus was not detected or was detected only in 1 of 3 mice.

To evaluate the protective activity of KPF1, C57BL/6 mice received increasing doses of KPF1 prior to a lethal intranasal challenge dose (10x MLD_50_) of pH1N1 (Fig. 5). All mice treated with PBS or IgG isotype control hmAb had severe weight loss and succumb to infection within 10 days p.i. (Fig. 5a and b). However, all mice treated with 10 mg/kg or 1 mg/kg of KPF1 maintained body weight and survived infection (Fig. 5a and b). Treatment with 0.1 mg/kg of KPF1 had minimal impact and non-significant differences on weight loss and survival (Fig. 5a and b). Consistent with increased survival, mice treated with 1 mg/kg or 10 mg/kg had significant reductions in viral titers in their lungs at two and four days p.i., including the absence of detectable virus in 2 of 3 mice treated with 10 mg/kg, suggestive of sterilizing immunity in these mice (Fig. 5c).

Given that KPF1 was able to neutralize other H1 influenza viruses *in vitro* using a microneutralization or HAI assay (Fig. 4 and Table 1), we assessed the *in vivo* protective activity of KPF1 against additional H1 influenza strains. For that, mice were treated with 10 mg/kg of IgG isotype control or with KPF1 and then challenged with a lethal dose (10x MLD_50_) of PR8, TX, or NC H1N1 influenza viruses^40,41^ and viral replication in lungs was analyzed (Fig. 5d). Correlating with the *in vitro* activity, mice that received the KPF1 hmAb were protected against the different viral challenges, with undetectable virus in mice treated with 10 mg/kg of KPF1 (Fig. 5d).

**Figure 6:**
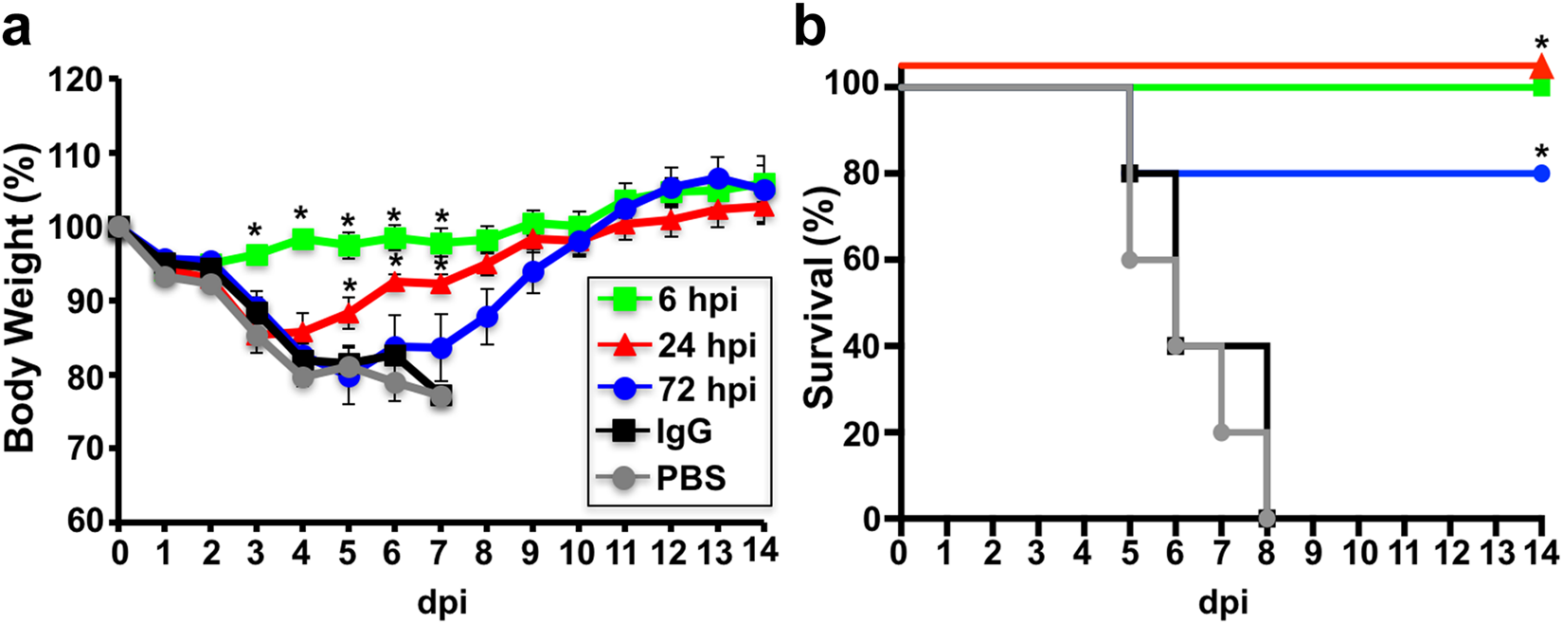
Therapeutic activity of KPF1 in infected mice. Female C57BL/6 mice (N = 5) were infected with 10x MLD_50_ of pH1N1 and then treated i.p. with 10 mg/kg of KPF1 hmAb at 6, 24 or 72 h p.i., or an isotype control hmAb (IgG isotype control; at 6 h p.i.) or PBS (at 6 h p.i.). Then, mice were monitored daily for 2 weeks for body weight loss (**a**) and survival (**b**). Mice that lost 25% of their body weight were sacrificed. Data represent the means ± SD (N = 5). *Indicates p < 0.05 (when the differences between mice treated with KPF1 hmAb and mice treated with PBS or IgG were significant using a one-tailed Student’s t test (body weight) or Mantel-Cox test (survival).

We next assessed the therapeutic activity of KPF1 (Fig. 6). To that end, mice were challenged with a lethal dose (10x MLD_50_) of pH1N1 and then treated 6 h, 24 h, or 72 h p.i. with 10 mg/kg KPF1. All mice treated with PBS or IgG isotype control hmAb had severe weight loss and succumbed to infection within 8 days p.i. (Fig. 6a and b). Early treatment (6 h p.i.) completely prevented weight loss and mortality, and delayed treatment at 24 h p.i. only resulted in only transient weight loss with all mice surviving the infection. Treatment with KPF1 as late as 72 h p.i. conferred 80% survival, with the surviving mice only having transient weight loss (Fig. 6a and b). Overall these results demonstrate that KPF1 has potent prophylactic and therapeutic activity against pH1N1 *in vivo*.

### Generation of *in vitro* escape mutants against KPF1

**Figure 7:**
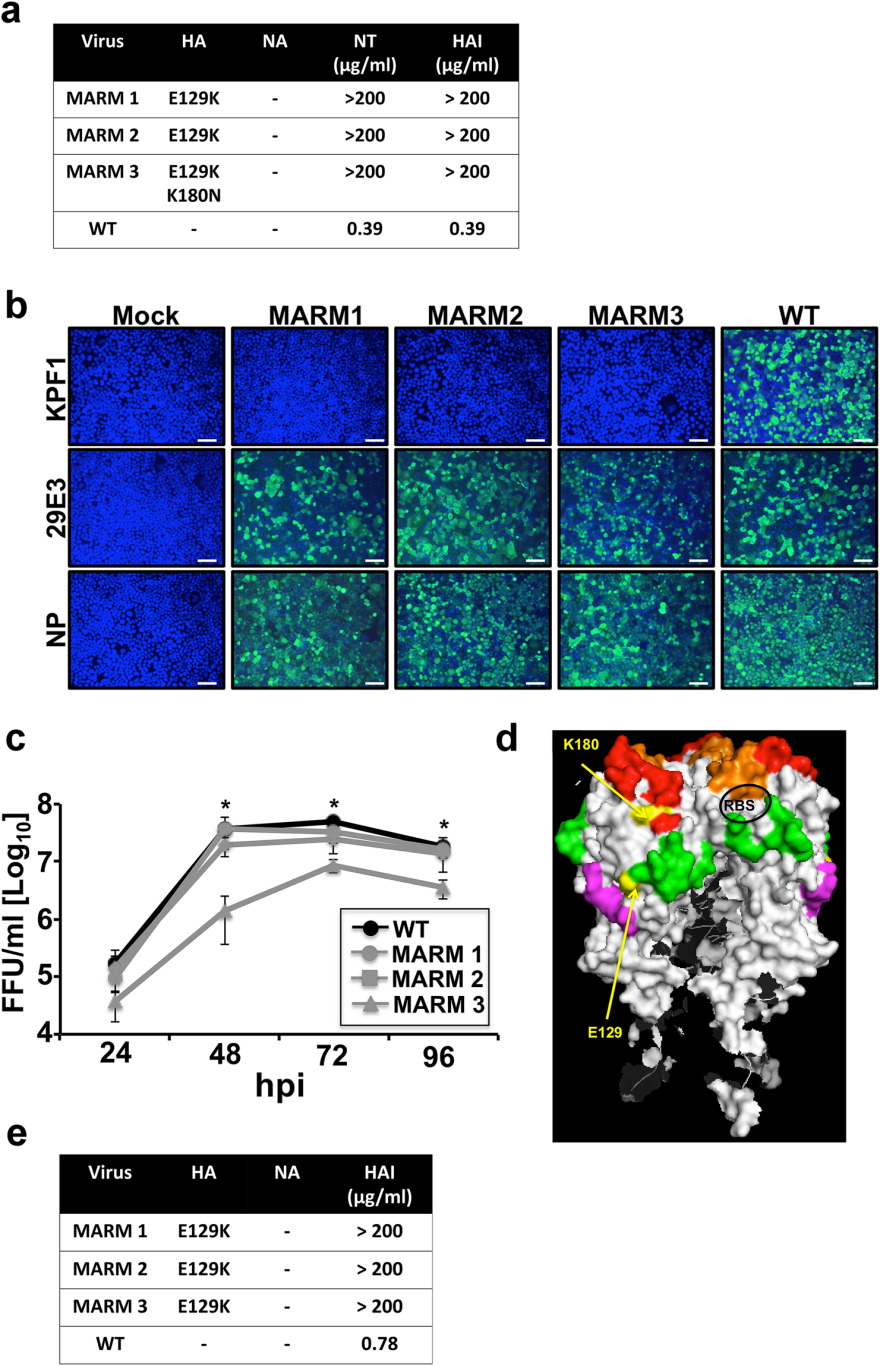
Generation and characterization of MARMs. (**a)** Amino acid mutations in the HA and NA of pH1N1 WT or mAb-resistant mutants (MARMs 1, 2 and 3) after 5 rounds of selection in the presence (MARMs) or absence (WT) of hmAb KPF1. The mutations effects on reactivity with the hmAb KPF1 were also evaluated in a microneutralization assay (NT_50_) and HAI. **(b**) Characterization of MARMs of pH1N1 by immunofluorescence. MDCK cells were mock infected (Mock) or infected (MOI 0.01) with pH1N1 WT or the MARMs (1, 2 and 3). At 36 h p.i., cells were fixed and protein expression was evaluated by IFA using the hmAb KPF1, or the mouse mAbs 29E3 (anti-HA) and HB-65 (anti-NP). DAPI was used for nuclear staining. Merge from representative images (10x magnification) are included. Scale bar, 50 nm. **(c**) Multicycle growth kinetics of pH1N1 WT and MARMs in MDCK cells. Virus titers in TCS of MDCK cells infected (MOI, 0.001) with pH1N1 WT or MARMs viruses were analyzed at the indicated h p.i by immunofocus assay (FFU/ml) using the anti-NP mouse mAb HB-65. Data represent the means ± SDs of the results determined for triplicate wells. *Indicates p < 0.05 (WT versus MARM 3) using a one-tailed Student’s t test. (**d**) Tridimensional protein structure for the globular head of HA of pH1N1. The image was created using the software program PyMol and the published structure for the HA of pH1N1 (3LZG^54^,). Positions of amino acid substitutions in the MARMs (E129 and K180) are colored in yellow. The residues at each antigenic site are colored as red for the Sa site, orange for the Sb site, green for the Ca site, and magenta for the Cb site. The receptor binding site (RBS) location in the structure is indicated. (**e**) Generation of MARMs for TX H1N1. Amino acid changes in the HA and NA of TX H1N1 WT or MARMs (1, 2 and 3) after 5 rounds of selection in the presence (MARMs) or absence (WT) of hmAb KPF1. The effect of E129K mutation on reactivity with KPF1 was also evaluated in a HAI assay, using WT TX H1N1 as an internal control.

**Figure 8:**
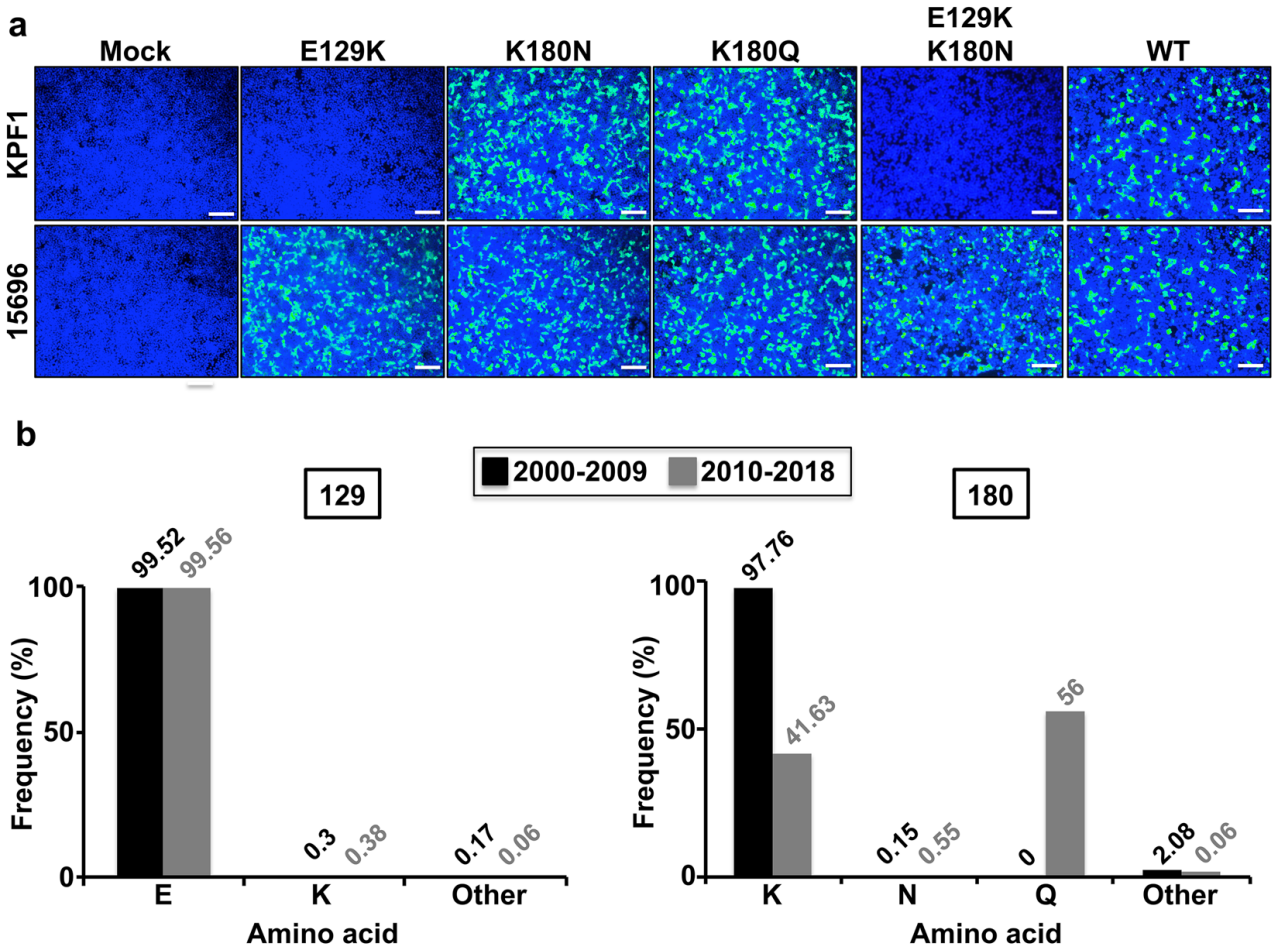
Relevance of amino acids 129 and 180 for the binding of KPF1 hmAb. (**a**) Binding of KPF1 hmAb to WT and mutant HA proteins. HEK293T cells were transiently transfected with the pCAGGS plasmids expressing WT or amino acid substitutions E129K, K180N, K180Q or E129K/K180N mutant HAs. Mock transfected cells were used as internal control. At 24 h post-transfection, cells were fixed and protein expression was evaluated by IFA using the hmAb KPF1, or a goat pH1N1 anti-HA polyclonal antibody as a control. DAPI was used for nuclear staining. Merge from representative images (10x magnification) are included. Scale bar, 50 nm. (**b**) Frequency of amino acid changes found in IAV H1N1 HA over time. Publicly available sequences of IAV H1N1 HA protein (Influenza Research Database) isolated between 2000–2009 (n = 8,586; black) or 2010–2018 (n = 8,417; grey) were analyzed and plotted according to the percentage of sequences containing the indicated amino acids at positions 129 (right) or 180 (left).

To identify amino acid residues that are critical for the formation of the KPF1 epitope we generated mAb-resistant mutants (MARMs) of pH1N1 (Fig. 7). WT pH1N1 virus was passaged in triplicate for five rounds in the presence of increasing concentrations of KPF1 and the NA and HA ORFs were sequenced from three MARMs (MARM1, MARM2, MARM3) (Fig. 7a). No mutations were detected in NA and all three MARMs shared a point mutation (E129K) located between the Ca and Cb antigenic sites. MARM3 also had an additional mutation (K180N), which is located within the Sa antigenic site (Fig. 7a and d). KPF1 did not bind to MARM1–3 as determined by immunofluorescence, although the binding to an NP-specific mAb and another pH1N1 head-specific HA mAb 29E3 was maintained (Fig. 7b). *In vitro* viral growth kinetics of MARM1 and MARM2 were comparable to that of pH1N1 WT, although the growth of MARM3 was significantly less (p < 0.05) than pH1N1 WT, suggesting that K180N mutation impacts viral fitness (Fig. 7c). Although the amino acids in the position 129 and 180 are far away from each other in the linear sequence of the HA protein, the analysis of the tridimensional protein structure shows that both amino acids are close in the folded HA protein (Fig. 7d). Interestingly, when TX H1N1 was propagated in the presence of KPF1, the same point mutation (E129K) identified for MARMs of pH1N1, was observed in all the MARMs of TX H1N1 (Fig. 7e). In addition, no mutations were identified in NA. These results confirm that E129 is important for the binding of KPF1 to HA. HAI assay was performed using pH1N1 or TX H1N1 viruses obtained from each round of virus selection. The HAI results indicate that MARMs are the majority viral population in passage 1 for pH1N1 or between passages 1 and 2 for TX H1N1 (data not shown). These results confirm that E129 is important for the binding of KPF1 to HA and suggest that KPF1 recognizes an epitope in the H1 HA globular head that dependent on residues near the Ca and Cb antigenic sites (E129) (Fig. 7d). Moreover, pCAGGS protein expression plasmids encoding the WT, single E129K, K180N, K180Q; or double E129K/K180N HA mutants were generated. Next, the ability of KPF1 to recognize the different HAs was evaluated by immunofluorescence assay in transfected 293 T cells (Fig. 8a). As was expected, KPF1 was unable to recognize HA proteins containing the amino acid change E129K (E129K and E129K/K180N) but was able to recognize HA proteins containing the amino acid change at position 180 (K180N and K180Q). This suggests that position 180 is not part of the KPF1 hmAb footprint and the amino acid change observed in 1 of 3 MARMS at position 180 was probably a random event or a compensatory mutation. In addition, an *in silico* analysis was performed to evaluate the frequency of amino acid residues E129 and K180 in more than 17,000 strains deposited in the database (https://www.fludb.org/brc/home.spg?decorator = influenza) of IAV H1N1 from 2000 to 2018 (Fig. 8b). Our findings indicate that amino acid E129 is highly conserved, since more than 99.5% of the analyzed IAV H1N1 sequences contain amino acid E at that position. Interestingly, the frequencies of other amino acids (including K, observed in our MARMs) represent less than 0.5% (Fig. 8b). This data suggests that this position is important for IAV H1N1 and therefore could be a suitable target for the development of antiviral therapies such as our described KPF1 hmAb. On the other hand, position 180 has higher variability, showing a shift from K to Q in the last decade (Fig. 8b). However, this variability does not affect the binding of KPF1 to HA.

## Discussion

Although currently licensed influenza vaccines and antivirals significantly mitigate the morbidity and mortality of influenza infections, they are sub-optimal and consequently substantial public health vulnerabilities exist in our ability to prevent and treat influenza. Improving the breadth of activity of both vaccines and antivirals to confer protection from emerging seasonal isolates and those with pandemic potential is a fundamental area of emphasis. In this study we have isolated and characterized KPF1, a hmAb which has broad activity against H1 influenza isolates and potent prophylactic and therapeutic activity *in vivo*, which is mediated by recognition of a conserved residue in the H1 hemagglutinin globular head.

KPF1 is highly specific to influenza H1 HA and recognized all H1 isolates tested with the exception of A/USSR/1977 (Fig. 3), likely due to the unique HA structure of this pandemic isolate^11,42^. The high potency of KPF1 is demonstrated *in vitro* by its neutralizing and HAI activities below 1 µg/ml (Fig. 4 and Table 1), and its high avidity and affinity (Fig. 2). Few hmAbs have been reported that have similar *in vitro* neutralizing activity of H1 influenza below 1 µg/ml^20,21,23,43–47^, highlighting the unique potency of KPF1.

The potent activity of KPF1 extends to its ability to protect and treat H1 infection *in vivo* (Figs 5 and 6). The challenge dose of 10x MLD_50_ pH1N1 used in this study exceeds by 2 to 5 fold that which has been used by others^48–53^ to evaluate the *in vivo* activity of mAbs against H1 influenza. With this high dose challenge of pH1N1, 1 mg/kg of KPF1 completely protected from infection in a prophylactic model (Fig. 5), and treatment with 10 mg/kg administered as late as 72 h p.i. significantly enhanced survival (Fig. 6). Remarkably, KPF1 was able to protect against the lethal challenge of multiple H1 influenza virus strains (Fig. 5). The true potential of KPF1 for treatment of influenza H1 infections may be underestimated as the dose of KPF1 or delay in treatment have not yet been further evaluated. Together these results suggest the *in vitro* and *in vivo* activity of KPF1 against H1 influenza is not bested to date by other mAbs.

KPF1 recognizes a highly conserved novel epitope (Fig. 8) in the HA1 globular head of H1 influenza strains that dependent on the E129 residue near the Ca and Cb antigenic sites (Fig. 7). The hmAb 2D1, which recognizes both 1918 and pH1N1 HA1, but has limited activity against most influenza H1 strains, recognizes an epitope centered on Sa which includes K180^54^, and selected for escape mutants at this residue^55^. 2D1 was not reported to interact with E129, suggesting the precise epitopes recognized by KPF1 are distinct than those of 2D1. The mouse mAb GC0587, which was generated from pH1N1 immunized mice is H1-specific and recognizes an epitope that contains both E129 and K180 residues^56^. However, its lack of reactivity against 1918 H1^56^ in contrast to KPF1, suggesting incomplete congruence in the epitopes recognized by both mAbs. Although viral neutralization is thought to be the major protective function of H1 specific Ab, the ability of H1 specific Abs to substantially contribute to Fc-mediated mechanisms of viral clearance such as Ab-dependent cell-mediated cytotoxicity (ADCC) and complement-dependent cytotoxicity (CDC) remains unclear^52,57,58^. Interestingly the E129 residue is located within an ADCC epitope that has been previously described^58^, suggesting evaluating the Fc-meditated activities of KPF1 are warranted. Overall, these results suggest the epitope recognized by KPF1 is broadly conserved in H1 strains, and recognition of this epitope can mediate neutralizing and HAI, and potentially ADCC activity. Further resolution of the epitope and assessment of its potential as an immunogen to induce broad protection from H1 influenza should be pursued.

The *in vitro* and *in vivo* activity profile of KPF1 as well as the E129 amino acid conservation, suggests it may have potential therapeutic value for the treatment and prevention of H1 influenza infections, which should be investigated further. A limitation of this current study is the evaluation of KPF1 therapeutic activity in only one animal model and against just a single H1 influenza isolate. Similarly, it is unclear if KPF1 acquired its breadth and potency as a result of the clonal evolution process, or if this is inherent from its germline. Thus, further experiments to evaluate its *in vivo* breadth and potency and linkage to somatic hypermutations are warranted. Although several hmAbs targeting HA stem epitopes have been identified that have broadly neutralizing activity against multiple influenza types and subtypes, their potency is commonly less than hmAbs targeting the HA globular head^39,59^, and their clinical efficacy has yet to be determined. Historically, influenza viruses causing pandemics have been subtype specific (e.g. H1N1, H2N2 or H3N2) and, therefore, having more potent globular head broadly neutralizing hmAbs rather than less specific and less potent neutralizing stalk reactive Abs may represent a better approach for pandemic preparedness. Accordingly, development of a cocktail of multiple high affinity type-specific hmAbs (including KPF1), collectively conferring universal breadth and protection through recognition of several epitopes may ultimately be an effective clinical therapeutic for influenza infection.

## Methods

### Cells and viruses

Canine Madin-Darby canine kidney (MDCK; ATCC CCL-34) and human embryonic kidney (HEK293T; ATCC CRL-11268) cells were grown at 37 °C with 5% CO_2_ in Dulbecco’s modified Eagle’s medium (DMEM; Mediatech, Inc.), 10% fetal bovine serum (FBS), and 1% PSG (penicillin, 100 units/ml; streptomycin 100 µg/ml; L-glutamine, 2 mM)^41^. The following influenza A and B viruses were propagated in MDCK cells: A/California/04/2009 H1N1 (pH1N1) wild-type (WT) and mCherry-expressing viruses^60,61^; A/Puerto Rico/08/1934 H1N1 (PR8 H1N1) WT and mCherry-expressing viruses^61^, A/Texas/36/91 H1N1 (TX H1N1), A/New Caledonia/20/99 H1N1 (NC H1N1), A/Wyoming/3/2003 H3N2 (H3N2) WT and mCherry-expressing viruses; and B/Brisbane/60/2008 (IBV) WT and mCherry-expressing viruses^60^. For infections, virus stocks were diluted in phosphate buffered saline (PBS), 0.3% bovine albumin (BA) and 1% PS (PBS/BA/PS). After viral infections, cells were maintained in post-infection (p.i.) medium, containing DMEM with 0.3% BA, 1% PSG, and 1 μg/ml TPCK-treated trypsin (Sigma)^62^. The virus titers of the stocks were determined by standard plaque assay (plaque forming units, PFU/ml) in MDCK cells as previously described^41^.

### Human monoclonal antibody (hmAb) generation

Peripheral blood was obtained from a healthy subject prior to, seven days, and one month after receiving the 2014–2015 seasonal inactivated quadrivalent influenza vaccine (A/California/07/2009 (H1N1) pdm09-like virus, A/Texas/50/2012 (H3N2)-like virus, B/Massachusetts/2/2012-like virus, B/Brisbane/60/2008-like virus) as standard-of-care at the University of Rochester Medical Center. The subject provided signed written informed consent. All procedures and methods were approved by the Research Subjects Review Board at the University of Rochester Medical Center, and all experiments were performed in accordance with relevant guidelines and regulations. PBMC and plasma was isolated using CPT tubes (Becton Dickinson, Franklin Lakes, NJ, USA). Fresh PBMC from seven days after immunization were stained for flow cytometry as previously described with anti-CD45-Qdot800 (HI30, Invitrogen, Carlsbad, CA), anti-CD19-APC-Cy7 (SJ25C1, BD Biosciences, San Jose, CA), anti-CD20-AlexaFluor 700 (2H7, Biolegend, San Diego, CA), anti-CD3-PacificOrange (UCHT1, Invitrogen), anti-IgD-FITC (IA6-2, BD), anti-CD27-Qdot655 (CLB-27/1, Invitrogen), anti-CD4-Qdot705 (S3.5, Invitrogen), anti-CD38-Qdot605 (HIT2, Invitrogen), anti-CD126-PE (M5, BD), and Live/Dead fixable aqua dead cell stain (Invitrogen). Plasmablasts were single cell sorted with a FACSAria (BD Biosciences) directly into 96-well PCR plates (Bio-Rad, Hercules, CA) containing 4 μL/well 0.5x PBS with 10 mM DTT (Invitrogen), and 8 U RiboLock (ThermoFisher) RNAse inhibitor. Plates were sealed with Microseal F Film (Bio-Rad) and immediately frozen at −80 °C until used for RT-PCR. cDNA was synthesized and semi-nested RT-PCR for IgH, Igλ, and Igκ V gene transcripts was performed as previously described^63^. Purified PCR products were sequenced at Genewiz Sequences and analyzed by IgBlast (www.ncbi.nlm.nih.gov/igblast) and IMGT/V-QUEST (www.imgt.org/IMGT_vquest) to identify germline V(D)J gene segments with highest identity and determine sequence properties. Expression vector cloning and transfection of human HEK293T cells were performed as previously described^63,64^. IgG was purified from culture supernatant using Magna Protein G beads (Promega, Madison, WI). 1069 D6 is a human IgG1 mAb that was used as an isotype control.

### Deep-sequencing immunoglobulin repertoire analysis

Using the sample collected 7 days after the subject received the 2014–2015 seasonal inactivated influenza vaccine, RNA was isolated from 1 × 10^7^ peripheral blood mononuclear cells (PBMC) using the RNeasy kit (Qiagen, Hilden, Germany), treated with DNase I (Turbo DNA-free Kit, Invitrogen, Vilnius, Lithuania) and used to synthesize cDNA with the qScript cDNA synthesis kit (QuantaBio, Beverly, MA). The resulting cDNA was used in subsequent PCR using Platinum Taq High Fidelity Polymerase (Invitrogen, Carlsbad, CA) and a touch-down PCR protocol starting with a 95 °C for 5 min of denaturing, then 2 cycles of 96 °C for 30 sec, 62 °C for 30 sec, and 68 °C for 1 min. The annealing temperature was dropped 2 °C for every other cycle until 55 °C which was used for the final 32 cycles. A final extension was performed at 68 °C for 10 min before holding at 12 °C. Degenerate primers were designed based on Sheid *et al*.^65^ with supplemental primers for VH2, IgM^66^, IgG^64^ and IgA (GAGGCTCAGCGGGAAGACCTTG). Forward and reverse primers also included a 12 nt index and the Illumina specific linker (forward: CAAGCAGAAGACGGCATACGAGATGTGACTGGAGT-TCAGACGTGTGCTCTTCCGATCT, reverse: AATGATACGGCGACCACCGAGATC-TACACTCTTTCCCTACACGACGCTCTTCCGATCT). Primers were synthesized and PAGE purified by Integrated DNA Technologies (Coralville, IA). Separate PCR reactions were completed for VH1/7, VH2, VH3, VH4, VH5, and VH6 using a final concentration of 0.05 mM of each forward primer and 0.25 mM of each reverse primer. Following PCR, products were resolved on a 1% agarose gel and bands corresponding to approximately 600 nt were excised. Bands were extracted using E.Z.N.A.^TM^ Gel Extraction Kit (Omega Bio-Tek, Norcross, GA) and all 6 VH reactions composited. PCR products were submitted to the University of Rochester Genomics Research Center where Qubit Fluorometric quantitation (ThermoFisher) and Bioanalyzer (Agilent Technologies, Santa Clara, CA) sizing, quantitation and quality control was performed prior to normalizing to 2 nM and flowcell hybridization and cluster generation for the MiSeq system (Illumina, Inc., San Diego, CA). Paired end reads (300 × 325 bp) were made.

Sequence analysis was performed using an in-house custom analysis pipeline described previously^67^. All sequences were aligned with IMGT.org/HighVquest^68^. KPF1 lineage tree was generated by identifying the lineage (identical VH, JH, HCDR3 length, and ≥85% HCDR3 similarity) containing the corresponding mAb sequence. Sequences with unique nucleotide VDJ re-arrangements (singletons) were removed as a conservative approach to avoid including diversity that may be a consequence of sequencing error, and resulting sequences were analyzed using Phylip’s protpars tool (version 3.695)^69^, turning on setting numbers 1, 4 and 5. The output file was then parsed using in-house custom scripts; collapsing any duplicate inferred sequences into an individual node and visualized using Cytoscape.

### Binding characterization

ELISA plates (Nunc Maxisorp, Thermo Fisher Scientific, Grand Island, NY) were coated with recombinant HA proteins (Protein Sciences, Meriden, CT) or RSV fusion (F) protein at 0.5 μg/mL, hMAbs were diluted in PBS, and binding detected with horseradish peroxidase (HRP)–conjugated anti-human IgG (Jackson ImmunoResearch, West Grove, PA). In select ELISAs increasing concentrations of urea was add to ELISA plate and incubated for 15 min at room temperature prior to detection with anti-IgG-HRP to evaluate avidity. The mPlex-Flu assay immunoglobulin quantification method was performed as previously described^37^. Assays were performed in 96 well black-walled microtiter-plates (Millipore, Billerica, MA). Just prior to assay, the coupled beads were vortexed for 15 sec and diluted to 50 beads of each bead region per μl and added at 25 μl beads per well. All plasma and hmAb dilutions and washes were performed using PBS (pH 7.2) containing 0.1% BSA (MP Biomedical, LLC, France) and 0.1% Brij-35 (Thermo Scientific, Waltham, MA). Twenty-five μl of diluted test plasma or hmAb were added to the 25 μl of beads in each well, in duplicate, and incubated at room temperature for 2 h on a rotary shaker (500 rpm) in the dark. Wells were then washed twice with 150 μl of wash buffer and 50 μl 1:400 diluted PE conjugated anti-human IgG (γ chain specific) specific secondary Ab (SouthernBiotech, Birmingham, AL) was added and the plates incubated for 2 h at room temperature on a rotary shaker (500 rpm) in the dark. After 3 additional washes, beads in each well were suspended with 100 μl Luminex driving solution (Luminex, Austin, TX) and analyzed on a Magpix multiplex reader (Luminex, Austin, TX), and results expressed as median fluorescence intensity (MFI).

### Binding affinity studies using surface plasmon resonance

The binding affinity of KPF1 to recombinant (r)HA of pH1N1 A/California/04/2009 (Protein Sciences Corp., Meriden, CT) was determined by SPR experiments performed with a Biacore T200 optical biosensor (Biacore, Uppsala, Sweden) at 25 °C. KPF1 (50 nM) was flowed across flow cell 2 of a Series S Sensor Chip Protein G (GE HealthCare, Uppsala, Sweden) using 60 sec contact time, 10 μl/min flow rate, and 30 sec for stabilization, capturing approximately 1800 resonance units (RU). Flow cell 1 was left blank (Protein G only) to serve as a reference. pH1N1 rHA was used to analyze binding with a 90 s contact time, 75 μl/m flow rate, and 700 sec dissociation time. Six different concentrations of the pH1N1 rHA in the range between 0.625–10 nM in two experiments were passed over each channel in a running buffer of PBS (pH 7.4) containing 0.05% Tween 20. After every binding event, the sensor surface was regenerated by repeated washes with 10 mM glycine (pH 1.5) at a flow rate of 30 µl/min. Each binding curve was analyzed after correcting for non-specific binding by subtraction of the signals obtained from the negative-control flow channel and buffer injections^70^. The kinetic parameters were obtained by local Rmax fitting due to the differing capture levels using the 1∶1 Langmuir interaction model within the Biacore T200 Evaluation Software 3.0 (GE Healthcare).

### Virus neutralization and fluorescence-based microneutralization assays

Virus neutralization assays were performed with WT and mCherry-expressing viruses as previously described^60,61^. Briefly, KPF1 hmAb or IgG1 isotype control hmAb were serially 2-fold diluted in PBS using 96-well plates (starting concentration of 200 µg/ml). One hundred PFUs of each virus were then added to the hmAb dilutions and incubated for 1 h at room temperature. MDCK cells (96-well plate format, 5 × 10^4^ cells/well, triplicates) were then infected with the hmAb-virus mixture for 1 h at room temperature. After viral adsorption, cells were maintained in p.i. medium, with 1 μg/ml TPCK-treated trypsin^62^ and incubated at 33 °C. For the fluorescence-based microneutralization assays, at 24–48 h p.i. cells were washed with PBS prior to red fluorescence quantification using a fluorescence plate reader (DTX-880, Becton Dickenson). Fluorescence values of mCherry virus-infected cells in the absence of hmAb were used to calculate 100% viral infection. Cells in the absence of viral infection were used to calculate the fluorescence background. Triplicate wells were used to calculate the mean and SD of neutralization. WT virus neutralization was determined by crystal violet staining at 48–72 h p.i. The neutralization titer 50 (NT_50_) was determined by a sigmoidal dose response curve (GraphPad Prism, v7.0).

### Evaluation of KPF1 for its prophylactic and therapeutic protective activities in mice

Five to seven-week-old female C57BL/6 mice were purchased from the National Cancer Institute (NCI) and maintained in the animal care facility at University of Rochester under specific pathogen-free conditions. All animal protocols were approved by the University of Rochester Committee of Animal Resources and complied with the recommendations in the Guide for the Care and Use of Laboratory Animals of the National Research Council^71^. For viral infections, mice were anesthetized intraperitoneally (i.p.) with 2,2,2-tribromoethanol (Avertin; 240 mg/kg of body weight) and inoculated intranasally (i.n.) with 10x the mouse lethal dose 50 (MLD_50_) of pH1N1 in a final volume of 30 µl. After viral infection, animals were monitored daily for morbidity (body weight loss) and mortality (survival). Mice showing more that 25% loss of body weight were considered to have reached the experimental endpoint and were humanely euthanized. To determine the prophylactic efficacy of KPF1, mice in groups of 11 were weighed and administered i.p. the hmAb at doses of 0.1, 1, and 10 mg/kg, or the irrelevant isotype control 1069 D6 hmAb at 10 mg/kg, or PBS. Twenty-four h after dosing, mice were infected intranasally (i.n.) with the indicated virus and monitored for two weeks (N = 5) (pH1N1). Viral replication (pH1N1, PR8 H1N1, TX H1N1, and NC H1N1) was determined by measuring viral titers in the lungs of infected mice at days 2 and 4 p.i. To that end, three mice from each group were euthanized and lungs were collected and homogenized. Mice were euthanized by administration of a lethal dose of avertin and exsanguination. Virus titers were determined by immunofocus assay (fluorescent focus-forming units, FFU/ml)^41,72^ using the anti-NP mAb HB-65 (ATTC) and a FITC-conjugated anti-mouse secondary Ab (Dako). For the study of therapeutic efficacy, mice groups (N = 5) at 6, 24 or 72 h p.i., were given i.p injections of 10 mg/kg of the KPF1 or isotype control IgG (at 6 h) or PBS (at 6 h). Geometric mean titers and data representation were performed using (GraphPad Prism, v7.0).

### Selection of monoclonal antibody-resistant mutants (MARMs)

All the MARMs were selected by incubating the pH1N1 or TX H1N1 influenza viruses under increasing concentrations of KPF1 hmAb. Briefly, MDCK cells were infected at low multiplicity of infection (MOI 0.01) with the pH1N1 in 24 well-plate. After 1 h of adsorption, p.i. medium containing the KPF1 hmAb was added to the wells. The plates were incubated at 33 °C for 2 to 3 days and observed daily for cytopathic effect (CPE). Once the infected cells exhibited more than 70% of CPE, tissue culture supernatants (TCS) were collected and used to infect fresh MDCK cells (MOI 0.01) as describe above. The selection protocol was repeated for five rounds with increasing concentrations (10, 25, 50, 100 and 100 µg/ml) of the KPF1 hmAb. MARMs cultures were performed in triplicate, and cultures without hmAb were maintained in parallel to control for cell-specific mutations.

### Plasmids

A polymerase I-driven pPolI plasmid containing the pH1N1 HA^73^ was used as template to introduce, using site-directed mutagenesis, the amino acid changes E129K, K180N, K180Q, and E129K/K180N. Then, the BbsI and PmlI restriction sites were used to subclone the HA fragment containing the amino acid substitutions into a polymerase II-driven pCAGGS protein expression plasmids^74^ using standard cloning techniques. Primers for the generation of the described mutants are available upon request. All plasmid constructs were verified by DNA sequencing (ACGT Inc.).

### Immunofluorescence assay (IFA)

For the characterization of the MARMs, confluent monolayers of MDCK cells were mock-infected or infected (MOI 0.01) with WT or the MARMs viruses. At 20 h p.i., cells were fixed with 4% PFA and permeabilized with 0.5% Triton X-100 in PBS for 15 min at room temperature. Cells were then incubated with hmAb KPF1, or with the pH1N1 HA mouse mAb 29E3^75^ or against NP (HB-65) for 1 h at 37 °C. After washing with PBS, cells were incubated with FITC-conjugated secondary anti-mouse Ab (Dako) and 4′,6-diamidino-2-phenylindole (DAPI; Research Organics) for 1 h at 37 °C. Cells were visualized and photographed using a fluorescence microscope (Olympus IX81) and camera (QIMAGING, Retiga 2000R) with a x10 objective. For the characterization of HA mutants, HEK293T cells were transiently transfected, using lipofectamine 2000, with 0.5 µg of the indicated pCAGGS plasmids and at 24 h post-transfection, HA expression was analyzed by IFA as indicated above, using the hmAb KPF1 or a goat pH1N1 anti-HA polyclonal antibody as internal control (BEI Resources NR-15696).

### Viral RT-PCR

Total RNA from infected MDCK cells was collected at 36 h p.i. and purified using TRIzol reagent (Invitrogen) according to the manufacturer’s specifications. cDNA synthesis for HA and NA mRNAs was performed using SuperScript® II Reverse Transcriptase (Invitrogen) and an oligo dT oligonucleotide (Invitrogen). Further, cDNAs were used as templates for PCR with primers specific for the viral HA and NA open reading frames (ORF). Then, the nucleotide sequences from MARMs and no-Ab control groups were determined (ACGT, Inc). Primer sequences for amplification of the pH1N1 HA and NA ORFs are available upon request.

### Virus growth kinetics

Multicycle virus growth kinetics were performed in confluent monolayers of MDCK cells (12-well plate format, 5 × 10^5^ cells/well, triplicates) infected (MOI 0.001) with the indicated viruses. Virus titers in TCS were determined by immunofocus assay (FFU/ml)^41,72^. Mean value and standard deviation (SD) were calculated using Microsoft Excel software.

### HAI assays

Hemagglutination inhibition (HAI) assays were used to determine the HA-neutralizing capability of KPF1. The assay was performed as describe previously^41,72^. Briefly, the KPF1 hmAb was serially diluted (2-fold) in 96-well V-bottom plates and mixed 1:1 with 4 hemagglutinating units (HAU) of pH1N1 for 60 min at room temperature. The HAI titers were determined by adding 0.5% turkey red blood cells (RBCs) to the virus-hmAb mixtures for 30 min on ice. The HAI titer was defined as the minimum amount of hmAb that completely inhibited hemagglutination.

### Data availability

The datasets generated during and/or analyzed during the current study are available from the corresponding author on reasonable request.
